# Implication of Human UGT2B7, 2B15, and 2B17 in 19-Norandrosterone Metabolism

**DOI:** 10.3389/fendo.2013.00075

**Published:** 2013-06-26

**Authors:** Emmanuel Strahm, Ulf Sjöberg, Mats Garle, Anders Rane, Lena Ekström

**Affiliations:** ^1^Division of Clinical Pharmacology, Karolinska Institutet, Stockholm, Sweden

**Keywords:** anabolic androgenic steroid, nandrolone, 19-norandrosterone, UGT2B7, UGT2B15

## Abstract

Nandrolone (19-nortestosterone) is an anabolic androgenic steroid commonly abused for doping purposes. Nandrolone is mainly metabolized in the liver into 19-norandrosterone prior to glucuronidation and excretion through urine over an extended period of time. Several UGTs (i.e., UGT2B7, UGT2B15, and UGT2B17) are thought to be the major enzymes responsible for conjugation of androgens in human. An *in vitro* study using recombinant enzymes expressed in insect cells showed that UGT1A4 and UGT2B7 are the two main enzymes responsible of 19-norandrosterone glucuronidation. However, the identity of the enzyme involved in nandrolone metabolism *in vivo* together with their relative contribution and regulation remain unknown. Inhibition assays using human liver microsomes (HLM) incubated with 19-norandrosterone and selective inhibitors confirmed that UGT2B7 and UGT2B15 are involved in 19-norandrosterone glucuronidation, since the presence of the specific UGT2B7 and UGT2B15 inhibitors gemfibrozil and valproic acid inhibited the 19-norandrosterone glucuronidation by 35 and 45%, respectively. HLM were genotyped for UGT2B15 D85Y, UGT2B7 H268Y, and the UGT2B17 deletion polymorphism. The glucuronidation activity on 19-norandrosterone was significantly higher in UGT2B15 DD than in the other UGT2B15 genotypes (*p* < 0.05). Moreover, human liver cancer HepG2 cells were exposed to androgens to determine if the transcriptional activity of the genes of interest was affected. Only UGT2B7 mRNA expression was significantly increased (1.8-folds) after incubation with nandrolone decanoate. These results show that the UGT2B7 and UGT2B15 are involved in 19-norandrosterone glucuronidation and that the UGT2B15 polymorphism (D85Y) is the only UGT genetic variation that influences the glucuronidation activity. This could partly explain the inter-individual variation in 19-norandrosterone excretion.

## Introduction

Nandrolone or 19-nortestosterone (19-NT) is an anabolic androgenic steroid (AAS) produced first for androgen replacement therapy as a testosterone analog. Due to its higher potency than testosterone itself it has been misused for doping purposes and forbidden in sports in 1976 (Hemmersbach and Grosse, 2010). Since then, and despite the appearance of other doping products, 19-NT preparations have remained one of the most abused substances in sports (WADA Adverse Analytical Findings and Atypical Findings, 2010, Montreal, Canada; www.wada-ama.org) as well as in the society (Eklof et al., 2003).

19-NT is mainly metabolized into 19-norandrosterone (19-NA) prior to glucuronidation and excretion via urine. Whereas 19-NT is known to be detectable in the serum for only 2–5 weeks after injection (Bagchus et al., 2005), urinary 19-NA in its glucuronidated form is detectable up to 1 year after injection of 19-NT and this excretion is prone to very large inter-individual variation (Kintz et al., 2001; Bagchus et al., 2005; Garevik et al., 2011).

Uridine 5′-diphospho-glucuronosyl transferases (UGTs) are known to be responsible for the final metabolism of many xenobiotics prior to their excretion in urine. Among the different UGTs family, UGT2B7, UGT2B15, and UGT2B17 are known to have a high capacity to conjugate androgens (Turgeon et al., 2001). A marked stereoselectivity in the activity of UGT enzymes was evidenced using recombinant enzyme assays. UGT2B7 and UGT2B17 catalyze the conjugation of androgens at the 3α-OH and the 17β-OH positions (Beaulieu et al., 1996; Jin et al., 1997; Coffman et al., 1998; Gall et al., 1999), whereas UGT2B15 has been reported to be a 17β-OH selective enzyme (Green et al., 1994; Kuuranne et al., 2003). To date, one study using selected UGT recombinants enzymes showed that 19-NA is mainly metabolized by UGT1A4 and UGT2B7 and, to a lesser extent, UGT1A1, UGT1A3, and UGT2B4 (Kuuranne et al., 2003).

Several genetic polymorphisms in UGTs have been reported to have an impact on their ability to metabolize androgens. A frequent polymorphism in UGT2B7 gene leading to a histidine to tyrosine substitution in codon 268 (H268Y) has shown variable functional impact with different substrates (Ritter et al., 1990; Jin et al., 1993; Coffman et al., 1998; Swanson et al., 2007). A relevant polymorphism leading to an aspartic acid to tyrosine substitution in codon 85 (D85Y) in the UGT2B15 gene was described (Chen et al., 1993; Levesque et al., 1997). The genotype YY was reported to be twice more active than DD on DHT that lead to a better protection against high androgen level and decrease the risk of prostate cancer (Levesque et al., 1997; MacLeod et al., 2000). A deletion polymorphism of the UGT2B17 gene has been identified (Murata et al., 2003; Wilson et al., 2004) and correlated to glucuroconjugation of testosterone (Jakobsson et al., 2006). A lack of this gene leads to a crucial decrease in urinary testosterone even after a testosterone injection (Schulze et al., 2008). However, there are no studies reporting UGT2B genetic variation and 19-NA glucuronidation activity.

The aim of this study was to identify the influence of UGT2B7, UGT2B15, and UGT2B17 genetic polymorphisms on 19-NA glucuronidation in order to better understand the large inter-individual variation in nandrolone elimination. Moreover, we investigated if supra-physiological doses of androgens affect the gene expression of UGT2B enzyme genes in HepG2 cells.

## Materials and Methods

### Liver samples

Fifty human liver tissue samples were obtained from human donors to our human liver bank (approved by the Ethics Review Board in Stockholm at Karolinska Institutet). The livers were homogenized in 50 mM potassium phosphate buffer (pH 7.4) and stored at −80°C until use. The human liver microsomes (HLM) were prepared according to standard procedure (von Bahr et al., 1980) and stored in 50 mM potassium phosphate buffer (pH 7.4) at -80°C until use. The protein concentration was determined according to Lowry et al. (1951).

### Genotyping

Genomic DNA from human liver homogenate was obtained using QIAmp DNA Blood Mini Kit from Qiagen (Hilden, Germany). UGT2B7 polymorphism (rs7439366) which alter the amino acid sequence Histidine (H) to Tyrosine (Y) at position 268 were investigated by 5′nuclease activity method, using upper primer AGCTGACGTATGGCTTATTCGAA and lower primer GGGTTTGGCAGGTTTGCA (Cybergen, Stockholm, Sweden) and probes FAM-TTCAGTTTCCATATCCAC and VIC-TTCAGTTTCCTCATCCACT (Applied Biosystems). UGT2B15 polymorphism (rs1902023) altering the amino acid sequence Tyrosine (Y) to Aspartic acid (D) at position 85 was investigated by 5′nuclease activity method using Taqman SNP Genotyping Assay C_–_27028164_10 from Applied Biosystems. The PCR reaction was carried out in 15 μL volume including 1–3 μL of genomic DNA, 2 × Taqman universal master mix, and run on an ABI 7500 Fast from Applied Biosystems. The PCR method consisted of an initial denaturation step at 95°C for 10 min followed by 40 cycles of denaturation for 15 s and annealing/elongation at 60°C for 1 min. The UGT2B17 deletion polymorphism was performed by real-time PCR analysis as described elsewhere (Schulze et al., 2008).

### Glucuronidation activity in human liver microsome and inhibition study

#### Chemicals

Methanol, n-pentane, potassium dihydrogenophosphate (KH_2_PO_4_), potassium hydrogenophosphate (K_2_HPO_4_), natrium carbonate (Na_2_CO_3_), magnesium chloride (MgCl_2_), and ethanethiol were provided by Merck (Darmstadt, Germany). Tris HCl, uridine 5′-diphospho-glucuronic acid (UDPGA) and natrium hydrogenocarbonate (NaHCO_3_) and ammonium iodide (NH4I), methyltestosterone (MeT), gemfibrozil, valproic acid, and testosterone enanthate (TE) were provided by Sigma-Aldrich Chemie GmbH (Munich, Germany). β-glucuronidase from *Escherichia coli* (*E coli*) was provided by Roche Diagnostics GmbH (Mannheim, Germany). N-methyl-N-(trimethylsilyl) trifluoroacetamide (MSTFA), were provided by Macherey-Nagel GmbH (Düren, Germany), Ultrapur water was obtained with a Milli-Q Reference Ultrapure Water Purification system equipped with a Q-Gard T1 purification pack and a BioPak Point-of-Use ultrafilter from Millipore (Billerica, MA, USA). Nandrolone decanoate (19-NTD), 19-norandrosterone (19-NA), and 19-norandrosterone glucuronide (19-NAG) were purchased from NMI (Pymble, Australia)-Hecogenin acetate was provided by Tokyo Chemical Industry Co. (Tokyo, Japan).

### Sample preparation

Human liver microsomes samples were thawed at 25°C and the volume equivalent to a final protein concentration of 1.0 mg/mL was spiked in a glass tube containing the 19-NA required for the assay. The final reaction volume of 50 μL was obtained by addition of Tris HCl 50 mM pH 7.4 containing UDGPA 20 mM and MgCl_2_ 50 mM. Inhibition assays were performed using specific UGTs inhibitors, hecogenin acetate at 10 μM for UGT1A4 inhibition, gemfibrozil 10 μM for UGT2B7, and valproic acid 1 mM for UGT2B15. After incubation during the required time at 37°C in a stirring bath, the reaction was stopped by addition of 2 mL n-pentane. A liquid-liquid extraction (LLE) with two times 2 mL n-pentane was performed to retrieve the free steroids. The organic phase was collected in a new glass tube containing 100 ng MeT as internal standard and evaporated to dryness under an air flow. Steroids were finally derivatized at 60°C for 30 min with 50 μL MSTFA/NH_4_I/Ethanethiol (10:0.02:0.03, v/w/v) prior to injection onto GC-MS. The aqueous phase was treated by addition of 0.5 mL phosphate buffer 1 M pH 7.0 and 70 μL β-glucuronidase from *E. coli* before incubation 60 min at 50°C. Then, 200 mg of solid carbonate buffer [Na_2_CO_3_/NaHCO_3_, 1:10 (w/w)] was added to reach a pH of 8.5–9.0. A LLE was performed using two times 2 mL n-pentane. The organic layer containing the deconjugated steroid was collected in a new glass tube containing 100 ng MeT as internal standard and evaporated to dryness under an air flow. Steroids were finally derivatized at 60°C for 30 min with 50 μL of MSTFA/NH4I/Ethanethiol prior to injection onto GC-MS.

### GC-MS analysis

The androgens quantification was performed on a gas chromatography system Agilent 6890 (Palo Alto, CA, USA) equipped with a column Agilent HP-1 (17 m × 0.2 mm I.D., 0.11 μm) and coupled to a mass analyzer Agilent 5973. A constant helium flow of 0.8 mL/min was applied on to the GC. The extracted sample (2 μL) was injected in the split mode (1:10). The temperatures were set at 250, 280, 150, and 230°C for the injector, transfer line, quadrupole, and ionization source, respectively. The GC oven temperature program was set as follow: hold 120°C for 1 min before gradient increase at 10°C/min to 200°C, then 2°C/min to 210°C, and finally at 40°C/min to 310°C and maintained 2 min before the next analysis. The ionization was performed at 70 eV. The compound identification was performed in the SCAN mode (*m/z* 50–500) with a scan time of 2.48 scan/s. The quantification was performed in the SIM mode with an analysis time of 30 ms/ion. Identity of 19-NA was controlled by the stability of the peak area ratios for *m/z* 405, 420, 315, 225, and 169. The ion with *m/z* 405 was corrected by MeT peak area (*m/z* 446) as internal standard and used for quantification.

### UGT2B mRNA expression in HepG2 cells after incubation with androgens

Human liver cancer HepG2 cells from ATCC (Manassas, VA, USA) were cultured in MEM supplemented with 10% fetal bovine serum from the same provider and maintained in an incubator in humidified atmosphere at 37°C and 5% CO_2_. Prior to androgen treatment, the HepG2 cells were split in 12-well plates and pre-incubated for 2–3 days. Nandrolone decanoate and TE were diluted in ethanol to 1 μM and added to the cells for 2 h prior to harvest using Trizol from Invitrogen (Carlsbad, CA, USA). The non-treated controls were incubated with vehicle only. The experiments were performed at eight independent times. Total RNA extraction from HepG2 cells was performed using 0.5 mL Trizol per well according to manufacturer’s instructions. RNA (0.5 μg) was reverse transcribed into cDNA with hexamer primer using first-strand cDNA synthesis kit from Invitrogen according to the manufacturer’s protocol and diluted 20 times in water.

The mRNA levels of UGT2B7, 2B15 and 2B17 in nandrolone treated HepG2s were determined by real-time PCR. Beta-actin (#4326315E) from Applied Biosystems was chosen as an endogenous housekeeping control gene. Quantitative real-time PCR was performed using the ABI 7500 Fast. Reaction mixtures contained SYBR green reaction mix from Kapa Biosystems (Woburn, MA, USA), UGTs primers from Cybergene for UGT2B7, UGT2B15, and UGT2B17 as described (Chouinard et al., 2006), 4 μL cDNA template in a total volume of 15 μL. Thermal cycling conditions included activation at 95°C for 10 min followed by 40 cycles of denaturation at 95°C for 15 s and annealing/elongation at 60°C for 1 min. Each reaction was performed in duplicate and no-template controls were included in each experiment. The untreated sample was employed as a calibrator and the delta CT-formula was used as described elsewhere (Schmittgen and Livak, 2008). The gene expression was quantified as the yield of the target gene relative to that of β-actin gene.

### Data analysis

Statistical analysis was performed using GraphPad Prism Software v 4.3 (San Diego, CA, USA). The genotype association analysis was calculated using one-way ANOVA and the mRNA expression levels in androgen and vehicle exposed cells were compared using Student’s *t*-test. Data are presented as mean ± SD.

## Results

### Human liver microsomes assays – inhibition studies

**Figure 1:**
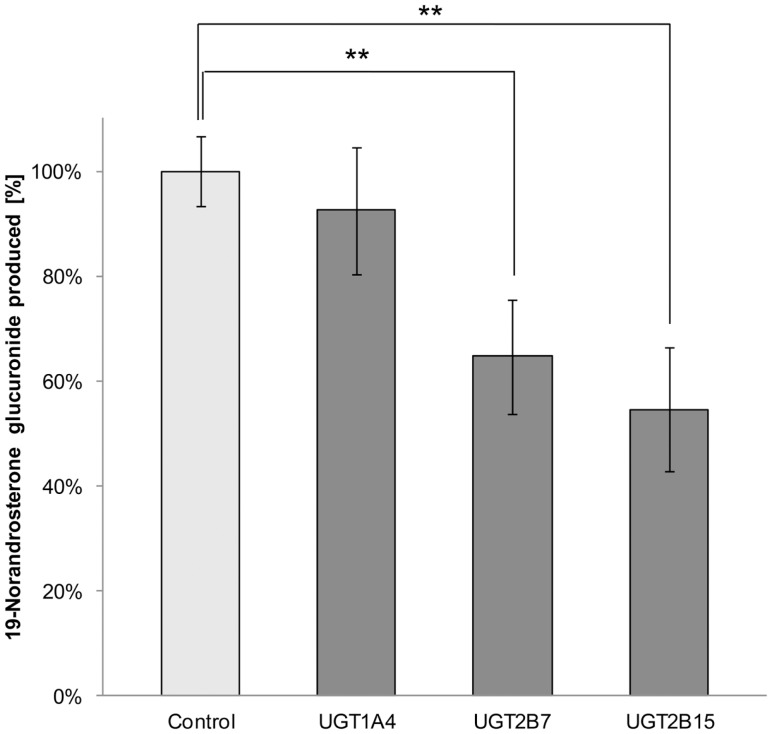
**Percentage of 19-norandrosterone glucuronide produced from 1 μM 19-norandrosterone in presence of UGTs specific inhibitors (hecogenin acetate 10 μM for UGT1A4, gemfibrozil 10 μM for UGT2B7, and valproic acid 1 mM for UGT2B15) in human liver microsomes (*n* = 5) presented as mean ± SD**. Significant inhibition was found for the UGT2B7 and UGT2B15 specific inhibitors.

Less than 1.5% of free 19-NA was detectable in 19-NAG solutions, and 19-NAG remained stable in the buffer without HLM when warmed at 37°C for 100 min. No increase of 19-NA concentration was observed. The inhibition assay showed a significant inhibition of 19-NAG production by 35 ± 11% for UGT2B7 (*p* = 0.04) and 45 ± 12% for UGT2B15 (*p* = 0.03) specific inhibitors valproic acid and gemfibrozil (Figure 1). No significant inhibition was observed with the UGT1A4 inhibitor hecogenin.

### Genetic variation and 19-NA glucuronidation

**Figure 2:**
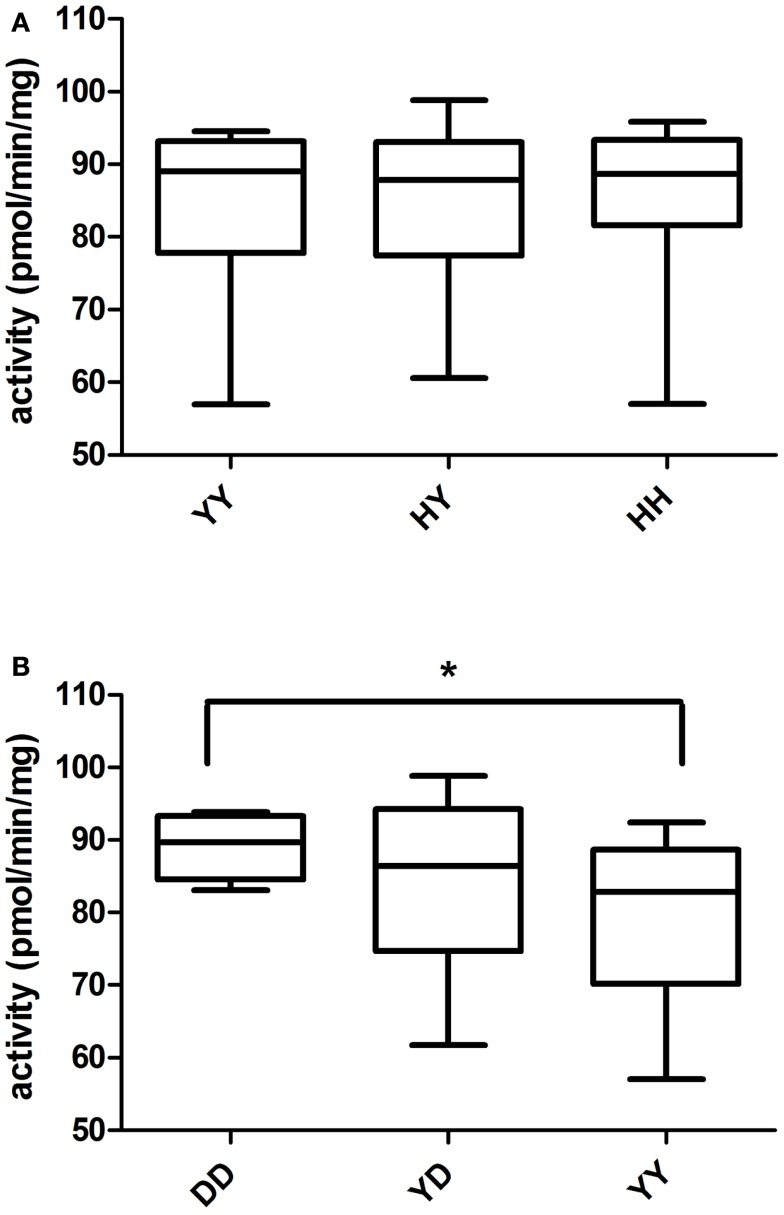
**Glucuronidation activity of 19-NA in human liver microsomes in relation to (A) UGT2B7 and (B) UGT2B15 polymorphisms**. There was no association between UGT2B7 (H268Y) genotype and glucuronidation activity, whereas a significant association between UGT2B15 (D85Y) polymorphism and glucuronidation activity (ANOVA *p* = 0.02) was observed.

UGT2B7 H268Y genotype was determined for 50 HLM prepared from our liver bank [YY (*n* = 12), YH (*n* = 25), HH (*n* = 13)]. The same HLM were incubated with 19-NA to determine their glucuronidation activity. No significant correlation between glucuronidation activity and UGT2B7 H268Y genotypes was revealed (Figure 2A). The same analysis was performed for UGT2B15 D85Y polymorphism. But due to problems during genotyping, only 42 of the HLM previously analyzed were genotyped [DD (*n* = 9), YD (*n* = 22), YY (*n* = 11)]. UGT2B15 DD homozygotes showed a significantly higher glucuronidation capacity than YD (*p* = 0.05) and YY (*p* = 0.02) subjects (Figure 2B). No significant difference in glucuronidation was observed between YD and YY genotypes. There was no significant difference between gender and UGTs glucuronidation activity.

Among all the HLM, nine microsomes of each UGT2B17 deletion polymorphism group (*del*/*del*, *ins*/*del, ins*/*ins*) were selected. No significant difference was reported between genotypes regarding glucuronidation activity (data not shown).

### UGTs mRNA expression in HepG2 cells

**Figure 3:**
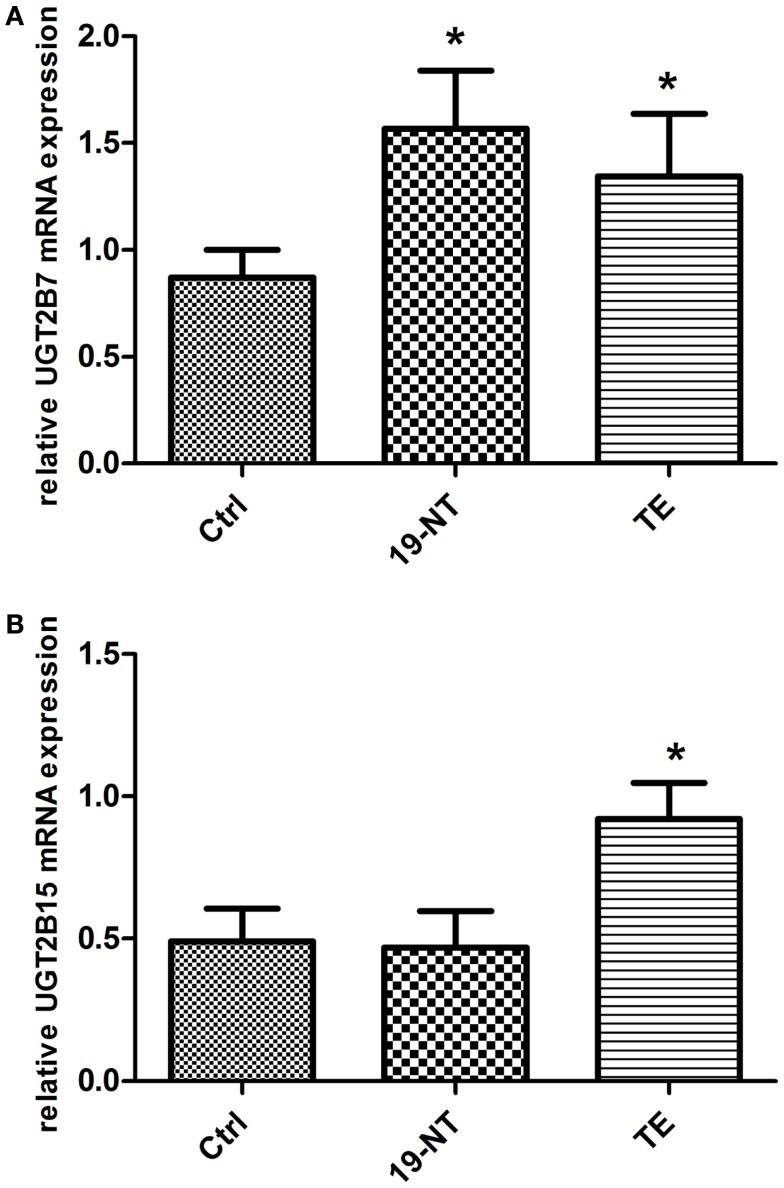
**(A)** UGT2B7 and **(B)** UGT2B15 mRNA expression in HepG2 cells after 2 h incubation with 1 μM 19-NT or testosterone enanthate (*n* = 8) presented as mean ± SD. UGT2B7 mRNA levels were significantly up-regulated by both androgens, whereas UGT2B15 was significantly induced by testosterone enanthate.

UGT2B7 mRNA expression was significantly increased in human liver HepG2 after incubation at 37°C for 2 h with 1 μM nandrolone decanoate (*p* = 0.02) and TE (*p* = 0.04) compared to incubation with the vehicle only (Figure 3A). UGT2B15 mRNA expression was significantly increased only after incubation with TE (*p* = 0.01) (Figure 3B).

## Discussion

The results of the inhibition study using UGT2B7 specific inhibitor gemfibrozil confirmed that this UGT is involved in the glucuronidation of the hydroxyl group in the 3α position of 19-NA. This is in line with *in vitro* observations from other studies (Jin et al., 1997; Coffman et al., 1998; Gall et al., 1999). However, the specific inhibition of the UGT1A4 enzyme by hecogenin acetate did not affect the HLM ability to glucuronidate 19-NA, which was previously suggested (Kuuranne et al., 2003). Surprisingly, UGT2B15, known to be involved in the glucuronidation of the 17β-OH position in androgens seems to be involved in 19-NA glucuronidation since its specific inhibitor, valproic acid, inhibited about 45% of the HLM glucuronidation activity.

The average glucuronidation activity in all tested HLM was of 84.5 ± 10.7 pmol/min/mg protein, that represents a glucuronidation of about 85% of the initial 19-NA added to the samples. This shows the high capacity of human liver microsome to conjugate 19-NA by glucuronidation. In addition to be excreted as glucuronides, 19-NA is also sulfated to some degree (Strahm et al., 2009) by cytosolic sulfotransferases.

A significantly higher glucuronidation activity was detected in individuals homozygous for the UGT2B15 D allele as compared to Y carriers. Information about the polymorphic variants of UGT2B15 activity toward androgens is conflicting. An early *in vitro* report indicated that the Y carriers show a higher activity than D carriers on dihydrotestosterone and androstane-3α, 17β-diol (Levesque et al., 1997), whereas another group reported that the D carriers have a higher glucuronidation activity than the Y carriers on the same androgens (Court et al., 2002).

No correlation with the polymorphism UGT2B7 H268Y was detected by us; which is in line with the results observed previously that UGT2B7 polymorphism did not influence the androsterone glucuronidation in HLM (Bhasker et al., 2000) and the serum level of androsterone glucuronide (Swanson et al., 2007). Androsterone is similar to 19-NA except with a methyl in the 19 position. As expected, UGT2B17 deletion polymorphism did not affect the 19-NA glucuronidation since this enzyme prefers glucuronidation of steroids at 17β-OH position and consequently plays an important role in testosterone glucuronidation (Jakobsson et al., 2006). As reported elsewhere for other substrates (Gallagher et al., 2010; Ekstrom et al., 2012) no sex differences in 19-NA glucuronidation activity was observed.

UGT2B7 mRNA expression was increased in HepG2 cells after incubation with 19-NT and TE whereas UGT2B15 mRNA expression was increased only after the incubation with TE. Our results indicate that androgens induce enzymes involved in its own elimination, which may further contribute to the inter-individual variability in disposition of 19-NA after the administration of nandrolone in healthy volunteers (Strahm et al., 2009) and in AAS abusers (Garevik et al., 2011). UGT2B17 on the other hand was not affected by androgen exposure of HepG2 cells. These results are opposite to what have been found in prostate cancer cells, where UGT2B15 and UGT2B17 has been shown to be down-regulated by androgens (Guillemette et al., 1997).

In conclusion, we show here that UGT2B7 and UGT2B15 are the main UGTs responsible for the glucuronidation of the main metabolite of nandrolone. However, only UGT2B15 D85Y polymorphism influences the glucuronidation of 19-norandrosterone with an activity significantly higher for the DD carriers. This could partly explain the inter-individual variation in 19-norandrosterone excretion, but a clinical study of nandrolone kinetics in healthy volunteers or studies in authentic nandrolone abusers would be required to confirm this hypothesis *in vivo*.

## Conflict of Interest Statement

The authors declare that the research was conducted in the absence of any commercial or financial relationships that could be construed as a potential conflict of interest.

## References

[B1] Bagchus W. M. Smeets J. M. Verheul H. A. De Jager-Van Der Veen S. M. Port A. Geurts T. B. (2005). Pharmacokinetic evaluation of three different intramuscular doses of nandrolone decanoate: analysis of serum and urine samples in healthy men. J. Clin. Endocrinol. Metab. 90, 2624–2630 10.1210/jc.2004-1526 15713722

[B2] Beaulieu M. Levesque E. Hum D. W. Belanger A. (1996). Isolation and characterization of a novel cDNA encoding a human UDP-glucuronosyltransferase active on C19 steroids. J. Biol. Chem. 271, 22855–22862 10.1074/jbc.271.37.22855 8798464

[B3] Bhasker C. R. McKinnon W. Stone A. Lo A. C. Kubota T. Ishizaki T. (2000). Genetic polymorphism of UDP-glucuronosyltransferase 2B7 (UGT2B7) at amino acid 268: ethnic diversity of alleles and potential clinical significance. Pharmacogenetics 10, 679–685 10.1097/00008571-200011000-00002 11186130

[B4] Chen F. Ritter J. K. Wang M. G. McBride O. W. Lubet R. A. Owens I. S. (1993). Characterization of a cloned human dihydrotestosterone/androstanediol UDP-glucuronosyltransferase and its comparison to other steroid isoforms. Biochemistry 32, 10648–10657 10.1021/bi00091a015 8399210

[B5] Chouinard S. Pelletier G. Belanger A. Barbier O. (2006). Isoform-specific regulation of uridine diphosphate-glucuronosyltransferase 2B enzymes in the human prostate: differential consequences for androgen and bioactive lipid inactivation. Endocrinology 147, 5431–5442 10.1210/en.2006-0229 16887906

[B6] Coffman B. L. King C. D. Rios G. R. Tephly T. R. (1998). The glucuronidation of opioids, other xenobiotics, and androgens by human UGT2B7Y(268) and UGT2B7H(268). Drug Metab. Dispos. 26, 73–77 9443856

[B7] Court M. H. Duan S. X. Guillemette C. Journault K. Krishnaswamy S. Von Moltke L. L. (2002). Stereoselective conjugation of oxazepam by human UDP-glucuronosyltransferases (UGTs): S-oxazepam is glucuronidated by UGT2B15, while R-oxazepam is glucuronidated by UGT2B7 and UGT1A9. Drug Metab. Dispos. 30, 1257–1265 10.1124/dmd.30.11.1257 12386133

[B8] Eklof A. C. Thurelius A. M. Garle M. Rane A. Sjoqvist F. (2003). The anti-doping hot-line, a means to capture the abuse of doping agents in the Swedish society and a new service function in clinical pharmacology. Eur. J. Clin. Pharmacol. 59, 571–577 10.1007/s00228-003-0633-z 13680032

[B9] Ekstrom L. Gök E. Johansson M. Garle M. Rane A. Schulze J. (2012). Doping and genetic testing: sex difference un UGT2B15 expression, testosterone glucuronidation activity and urinary testosterone/epitestosterone glucuronide ratio. Curr. Pharmacogenomics Person. Med. 10, 226–230

[B10] Gall W. E. Zawada G. Mojarrabi B. Tephly T. R. Green M. D. Coffman B. L. (1999). Differential glucuronidation of bile acids, androgens and estrogens by human UGT1A3 and 2B7. J. Steroid Biochem. Mol. Biol. 70, 101–108 10.1016/S0960-0760(99)00088-6 10529008

[B11] Gallagher C. J. Balliet R. M. Sun D. Chen G. Lazarus P. (2010). Sex differences in UDP-glucuronosyltransferase 2B17 expression and activity. Drug Metab. Dispos. 38, 2204–2209 10.1124/dmd.110.035345 20810538 PMC2993461

[B12] Garevik N. Strahm E. Garle M. Lundmark J. Stahle L. Ekstrom L. (2011). Long term perturbation of endocrine parameters and cholesterol metabolism after discontinued abuse of anabolic androgenic steroids. J. Steroid Biochem. Mol. Biol. 127, 295–300 10.1016/j.jsbmb.2011.08.005 21884791

[B13] Green M. D. Oturu E. M. Tephly T. R. (1994). Stable expression of a human liver UDP-glucuronosyltransferase (UGT2B15) with activity toward steroid and xenobiotic substrates. Drug Metab. Dispos. 22, 799–805 7835232

[B14] Guillemette C. Levesque E. Beaulieu M. Turgeon D. Hum D. W. Belanger A. (1997). Differential regulation of two uridine diphospho-glucuronosyltransferases, UGT2B15 and UGT2B17, in human prostate LNCaP cells. Endocrinology 138, 2998–3005 10.1210/en.138.7.2998 9202245

[B15] Hemmersbach P. Grosse J. (2010). Nandrolone: a multi-faceted doping agent. Handb. Exp. Pharmacol. 195, 127–154 10.1007/978-3-540-79088-4_6 20020363

[B16] Jakobsson J. Ekstrom L. Inotsume N. Garle M. Lorentzon M. Ohlsson C. (2006). Large differences in testosterone excretion in Korean and Swedish men are strongly associated with a UDP-glucuronosyl transferase 2B17 polymorphism. J. Clin. Endocrinol. Metab. 91, 687–693 10.1210/jc.2005-1643 16332934

[B17] Jin C. Miners J. O. Lillywhite K. J. Mackenzie P. I. (1993). Complementary deoxyribonucleic acid cloning and expression of a human liver uridine diphosphate-glucuronosyltransferase glucuronidating carboxylic acid-containing drugs. J. Pharmacol. Exp. Ther. 264, 475–479 8423545

[B18] Jin C. J. Mackenzie P. I. Miners J. O. (1997). The regio- and stereo-selectivity of C19 and C21 hydroxysteroid glucuronidation by UGT2B7 and UGT2B11. Arch. Biochem. Biophys. 341, 207–211 10.1006/abbi.1997.9949 9169006

[B19] Kintz P. Cirimele V. Dumestre-Toulet V. Ludes B. (2001). Doping control for nandrolone using hair analysis. J. Pharm. Biomed. Anal. 24, 1125–1130 10.1016/S0731-7085(00)00570-7 11248508

[B20] Kuuranne T. Kurkela M. Thevis M. Schanzer W. Finel M. Kostiainen R. (2003). Glucuronidation of anabolic androgenic steroids by recombinant human UDP-glucuronosyltransferases. *Drug* Metab. Dispos. 31, 1117–1124 10.1124/dmd.31.9.1117 12920167

[B21] Levesque E. Beaulieu M. Green M. D. Tephly T. R. Belanger A. Hum D. W. (1997). Isolation and characterization of UGT2B15(Y85): a UDP-glucuronosyltransferase encoded by a polymorphic gene. Pharmacogenetics 7, 317–325 10.1097/00008571-199708000-00007 9295060

[B22] Lowry O. H. Rosebrough N. J. Farr A. L. Randall R. J. (1951). Protein measurement with the Folin phenol reagent. J. Biol. Chem. 193, 265–275 14907713

[B23] MacLeod S. L. Nowell S. Plaxco J. Lang N. P. (2000). An allele-specific polymerase chain reaction method for the determination of the D85Y polymorphism in the human UDP-glucuronosyltransferase 2B15 gene in a case-control study of prostate cancer. Ann. Surg. Oncol. 7, 777–782 10.1007/s10434-000-0777-3 11129427

[B24] Murata M. Warren E. H. Riddell S. R. A. (2003). human minor histocompatibility antigen resulting from differential expression due to a gene deletion. J. Exp. Med. 197, 1279–1289 10.1084/jem.20030044 12743171 PMC2193779

[B25] Ritter J. K. Sheen Y. Y. Owens I. S. (1990). Cloning and expression of human liver UDP-glucuronosyltransferase in COS-1 cells. 3,4-catechol estrogens and estriol as primary substrates. J. Biol. Chem. 265, 7900–7906 2159463

[B26] Schmittgen T. D. Livak K. J. (2008). Analyzing real-time PCR data by the comparative C(T) method. Nat. Protoc. 3, 1101–1108 10.1038/nprot.2008.73 18546601

[B27] Schulze J. J. Lundmark J. Garle M. Skilving I. Ekstrom L. Rane A. (2008). Doping test results dependent on genotype of uridine diphospho-glucuronosyl transferase 2B17, the major enzyme for testosterone glucuronidation. J. Clin. Endocrinol. Metab. 93, 2500–2506 10.1210/jc.2008-0218 18334593

[B28] Strahm E. Baume N. Mangin P. Saugy M. Ayotte C. Saudan C. (2009). Profiling of 19-norandrosterone sulfate and glucuronide in human urine: implications in athlete’s drug testing. Steroids 74, 359–364 10.1016/j.steroids.2008.11.005 19056413

[B29] Swanson C. Lorentzon M. Vandenput L. Labrie F. Rane A. Jakobsson J. (2007). Sex steroid levels and cortical bone size in young men are associated with a uridine diphosphate glucuronosyltransferase 2B7 polymorphism (H268Y). J. Clin. Endocrinol. Metab. 92, 3697–3704 10.1210/jc.2007-0530 17579197

[B30] Turgeon D. Carrier J. S. Levesque E. Hum D. W. Belanger A. (2001). Relative enzymatic activity, protein stability, and tissue distribution of human steroid-metabolizing UGT2B subfamily members. Endocrinology 142, 778–787 10.1210/en.142.2.778 11159850

[B31] von Bahr C. Groth C. G. Jansson H. Lundgren G. Lind M. Glaumann H. (1980). Drug metabolism in human liver in vitro: establishment of a human liver bank. Clin. Pharmacol. Ther. 27, 711–725 10.1038/clpt.1980.102 6769631

[B32] Wilson W. III Pardo-Manuel de Villena F. Lyn-Cook B. D. Chatterjee P. K. Bell T. A. Detwiler D. A. (2004). Characterization of a common deletion polymorphism of the UGT2B17 gene linked to UGT2B15. Genomics 84, 707–714 10.1016/j.ygeno.2004.06.011 15475248

